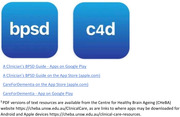# Evidence‐based comprehensive overview of research quality, effectiveness of interventions, and practice‐based principles for clinicians supporting people who present with changed behaviours associated with dementia and for family care partners

**DOI:** 10.1002/alz.088426

**Published:** 2025-01-09

**Authors:** Kim Burns, Anne‐Nicole S. Casey, Henry Brodaty

**Affiliations:** ^1^ Centre for Healthy Brain Ageing (CHeBA), University of New South Wales (UNSW) Sydney, Sydney, NSW Australia; ^2^ University of New South Wales (UNSW), Sydney, NSW Australia

## Abstract

**Background:**

Guidelines that provide current and comprehensive overviews of the evidence quality and effectiveness of interventions that address behaviours and psychological symptoms associated with dementia (BPSD, also known as Changed Behaviours) are needed for clinicians, professional care staff and family care partners. With funding provided by the Australian Government Department of Health and Aged Care, we aimed to update the existing *Behaviour Management: A Guide to Good Practice, Managing Behavioural and Psychological Symptoms of Dementia* (2012) text and app resources to reflect findings from the most recent literature and other sources.

**Methods:**

We systematically searched PubMed, Medline, Embase, and PsycINFO for psychosocial and environmental and biological and pharmacological interventions published between 2012‐2021 that addressed BPSD. We reviewed identified studies, rated research quality using predetermined criteria, calculated effect sizes where possible, and synthesised the moderate to strong quality evidence and advice from the most recent clinical guidelines. The final updated resource was reviewed by frontline expert advisors and dementia support specialists working with people with lived experience.

**Results:**

We identified 420 studies of psychosocial and environmental interventions and 221 studies of biological/pharmacological interventions for review and rating. The final updated resource *A Clinician’s BPSD Guide 2023: Understanding and helping people experiencing changed behaviours and psychological symptoms associated with dementia* includes moderate to strong quality evidence from 348 studies of psychosocial and environmental interventions and 178 studies of biological/pharmacological interventions. Sections detailing additional considerations for Aboriginal and Torres Strait Island peoples and those from culturally and linguistically diverse backgrounds with dementia are included.

**Conclusions:**

Published research evidence relating to interventions to support people living with dementia who experience changed behaviours has increased dramatically in volume and quality since 2012. Future advances will necessitate ongoing evidence updates. The *2023 Clinician’s BPSD Guide* provides a comprehensive overview of evidence and practice‐based principles for supporting people who present with behaviours and psychological symptoms associated with dementia (BPSD) and their family care partners. Travel size summary versions *A Clinician’s Field Guide 2023* and *A Guide for Carers 2023*, and mobile apps *A Clinician’s BPSD Guide* and *CareForDementia* (c4d) are also available. All are free to download^1^.